# Eotaxin-2 and eotaxin-3 in malaria exposure and pregnancy

**DOI:** 10.1186/s12936-022-04372-7

**Published:** 2022-11-15

**Authors:** Cristina Mancebo-Pérez, Marta Vidal, Ruth Aguilar, Diana Barrios, Azucena Bardají, Maria Ome-Kaius, Clara Menéndez, Stephen J. Rogerson, Carlota Dobaño, Gemma Moncunill, Pilar Requena

**Affiliations:** 1 ISGlobal, Hospital Clínic- Universitat de Barcelona, Barcelona, Catalonia Spain; 2 Manhiça Health Research Center (CISM), Manhiça, Mozambique; 3 Consorcio de Investigación Biomédica en Red de Epidemiología Y Salud Pública (CIBERESP), Barcelona, Spain; 4 Department of Infectious Diseases, University of Melbourne, the Doherty Institute, Melbourne, Australia; 5 Papua New Guinea Institute of Medical Research, Goroka, Papua New Guinea; 6 Consorcio de Investigación Biomédica en Red de Enfermedades Infecciosas (CIBERINFEC), Barcelona, Spain; 7 Department of Preventive Medicine and Public Health, Universidad de Granada, Granada, Spain; 8 Instituto de Investigación Biosanitaria (Ibs.GRANADA), Granada, Spain; 9 Present Address: Infectious Diseases Department, Hospital Universitari Vall d’Hebron, Institut de Recerca (VHIR), Universitat Autònoma de Barcelona, Barcelona, Spain

**Keywords:** Eotaxin, Chemokine, Malaria, Placenta, Pregnancy

## Abstract

**Background:**

Eotaxin-1 concentrations in plasma have been inversely associated with malaria exposure, malaria infection and pregnancy, but the effect of these conditions on the levels of the related chemokines eotaxin-2 and eotaxin-3 remains unknown.

**Methods:**

Eotaxin-2 and -3 concentrations were measured in 310 peripheral or placental plasma samples from pregnant and non-pregnant individuals from Papua New Guinea (malaria-endemic country) and Spain (malaria-naïve individuals) with previous data on eotaxin-1 concentrations. Correlations between eotaxin concentrations were examined with the Spearman’s test. Differences in eotaxin concentrations among groups were evaluated with the Kruskal–Wallis or Mann Whitney tests. The pairwise Wilcoxon test was performed to compare eotaxin-2 concentration between peripheral and placental matched plasmas. Univariable and multivariable linear regression models were estimated to assess the association between eotaxins and *Plasmodium* infection or gestational age.

**Results:**

Eotaxin-2 concentrations in plasma showed a weak positive correlation with eotaxin-3 (rho = 0.35, p < 0.05) concentrations. Eotaxin-2 concentrations in the malaria-exposed non-pregnant group were significantly lower than the in the malaria-naive non-pregnant and the malaria-exposed pregnant groups. Eotaxin-3 plasma concentrations were lower in malaria-exposed than in non-exposed groups (p < 0.05), but no differences were found associated to pregnancy. Eotaxin-2 and eotaxin-3 plasma concentrations were negatively correlated with anti-*Plasmodium* IgG levels: PfDBL5ε-IgG (rho_Eo2_ = − 0.35, p = 0.005; rho_Eo3_ =− 0.37, p = 0.011), and eotaxin-3 was negatively correlated with PfDBL3x-IgG levels (rho_Eo3_ =− 0.36; p = 0.011). Negative correlations of eotaxin-2 and 3 in plasma were also observed with atypical memory B cells (rho_Eo2_ = − 0.37, p < 0.001; rho_Eo3=_ − 0.28, p = 0.006), a B cell subset expanded in malaria-exposed individuals. In addition, a borderline negative association was observed between eotaxin-3 concentrations and *Plasmodium* infection (adjusted effect estimate, β = − 0.279, 95% CI − 0.605; 0.047, p = 0.091). Moreover, eotaxin-2 placental concentrations were significantly increased compared to peripheral concentrations in the malaria-exposed pregnant group whereas the contrary was observed in the non-exposed pregnant group (p < 0.005).

**Conclusion:**

Although a clear epidemiological negative association is observed between eotaxins concentrations and malaria exposure and/or infection, pregnancy may alter this association for eotaxin-2. Further research is required to understand the role of these chemokines in this disease and in combination with pregnancy.

## Background

The mechanisms involved in naturally acquired immunity (NAI) to malaria are not completely understood, but it is well accepted that they include both the cellular and humoral arms of the immune system [1, 2]. Of note, a subset of memory B cells (MBC) with an atypical phenotype (CD27^−^) has been repeatedly found to be expanded in individuals with large malaria exposure, including cohorts of pregnant women [3–6]. These atypical MBC seem to produce neutralizing antibodies against *Plasmodium falciparum* [7], but their impact on antibody-mediated immunity to malaria remains unclear. Moreover, marginal zone-like memory B cells (MZ-MBC) frequencies have been shown to be decreased in malaria-exposed individuals [6, 8], but its impact on NAI also needs to be elucidated.

Pregnant women have increased susceptibility to malaria infection and disease, a condition known as malaria in pregnancy (MiP) [9]. The immune response is modified during gestation in order to tolerate the presence of the fetus [6, 10–14], with a bias towards a Th2 response which may contribute to the increased susceptibility and severity of infections during pregnancy [15]. However, it is broadly accepted that a pro-inflammatory response is also essential for some pregnancy key processes like implantation or parturition. The role of many other biomarkers outside the classical inflammation-Th1-Th2 cytokine frame in MiP remains to be determined.

In a previous study, pregnancy and malaria exposure were associated with decreased plasma concentrations of the chemokine eotaxin-1 (CCL11), and its levels showed the highest (negative) correlation among thirty biomarkers with atypical MBC frequencies in a cohort from Papua New Guinea [6]. Furthermore, eotaxin-1 plasma concentrations were negatively associated with *Plasmodium vivax* infection during pregnancy and positively associated with haemoglobin levels at delivery in a multicenter study [15]. Additionally, studies in non-pregnant individuals have shown negative associations between plasma eotaxin-1 and malaria exposure, and with IgG plasma levels against the malarial antigen apical membrane antigen 1 (AMA-1; unpublished results). Moreover, a negative correlation between *P. falciparum* parasitaemia and eotaxin-1 plasma concentrations was found in two different cross-sectional studies performed in years of different malaria transmission intensity [16]. Interestingly, a significant increase (instead of decrease) of eotaxin-1 levels has been reported in plasma from patients with other infectious diseases like dengue, *Clostridium difficile* infection*,* tuberculosis or HIV [17–21].

Eotaxin-1 concentrations seem to be also altered during pregnancy. One study has demonstrated that eotaxin-1 plasma levels decrease significantly during pregnancy, with the lowest found in the third trimester, and that these recover post-partum (after 6 weeks) [13]. The same result was observed in a cohort of pregnant women living in the tropics. Moreover, in that cohort, eotaxin 1 concentrations in the placenta was even lower than in peripheral blood at delivery [22].

Eotaxin-1 is a potent eosinophil chemoattractant involved in inflammatory diseases as well as parasitic infections [23]. Eotaxin-2 (CCL24) and eotaxin-3 (CCL26) are less well-studied, but they also stimulate eosinophil chemotaxis [24] and the three share the same receptor, CCR3, expressed selectively on eosinophils, basophils, mast cells, some Th2 cells and even in atypical MBC [6, 25–27]. The peripheral levels of eotaxin-2 and -3 have not been studied during pregnancy nor in malaria cohorts or any other infectious diseases.

The aim of the present study was to determine the plasma concentrations of eotaxin-2 and eotaxin-3 in malaria-exposed pregnant and non-pregnant individuals as well as in malaria-naive pregnant and non-pregnant individuals, and to determine their associations with pregnancy and malaria infection/exposure, as well as other parameters known to be affected by malaria exposure.

## Methods

### Study design and population

This study includes peripheral and placental plasma samples from pregnant and non-pregnant subjects from Papua New Guinea (PNG) who participated in the PregVax study [28] (FP7-HEALTH-201588). PNG is characterized by high levels of transmission of both *P*. *falciparum* and *P. vivax,* but transmission was much decreased in the period of recruitment of this cohort [28]. Peripheral blood samples from pregnant women obtained at recruitment (first antenatal visit occurring at any time during pregnancy except labor) or at delivery were used. Only 10 of the samples were matched. Malaria infection was assessed by microscopy and/or real-time polymerase chain reaction (PCR) as previously reported [28], and was defined as a positive smear and/or PCR for any *Plasmodium* species.

To achieve the aims of this study, peripheral and placental plasma samples (n = 255) from PregVax subjects from whom there was available data on other cytokine concentrations were used: 54 peripheral plasma samples from malaria-exposed pregnant women at enrolment (EP-e), 91 peripheral and 72 placental-matched samples at delivery (EP-d and EP-p, respectively) and 38 peripheral plasma samples from non-pregnant exposed women (ENP). In addition, peripheral plasma samples from malaria-naïve non-pregnant (NNP, n = 23) as well as peripheral and placental-matched samples from malaria-naïve pregnant women (NP, n = 16 and NP-p n = 16) were taken from volunteers resident in Spain who had never travelled to malaria-endemic areas. Matched peripheral-placental blood samples were only analysed for eotaxin-2, based on the hypothesis explained in the results section.

### Quantification of cytokines

Eotaxin-2 and eotaxin-3 concentrations were quantified using the enzyme-linked immunosorbent assay (ELISA) sandwich technique, with the following commercial kits: ‘Human CCL24/Eotaxin-2/MPIF-2’ kit (DY343) from R&D Systems (Madrid, Spain), and ‘Human Eotaxin-3 (CCL26) Standard ELISA Development’ kit (900-K167) from PeproTech (London, UK). Samples were tested directly or after dilution, depending on the group of subjects. Assays were conducted according to manufacturer’s instructions, with the exception of eotaxin-2 ELISA in which half of the volume of each reagent was used to reduce the amount of plasma used without diluting it. Several tests were performed before the analysis to assess the sensitivity of the test under these conditions. Finally, the optical density was measured in an EPOCH instrument and the values fitted into a 4-parameter logistic regression curve automatically calculated by the GEN5 software, using the standards provided in the kit. Only eotaxin-3 measurements from 116 of all peripheral plasmas were available due to plasma volume limitations. Eotaxin-1 concentrations were previously determined in the same cohort by the Luminex technology using the Invitrogen™ Cytokine Magnetic 30-Plex Panel kit (Thermo Fisher Scientific, Madrid, Spain) [15, 22].

### Quantification of antibodies

The malaria-specific IgG levels were measured in a previous study using the Luminex technology with an antigen panel developed in-house [6] and results are expressed as median fluorescence intensity (MFI). *Plasmodium* antigens included in the panel were PfMSP-1_19_, PfEBA175, PfDBL3X, PfDBL5ε, PfDBL6ε, PfAMA-1, PvLP1 and PvLP2, three *P. vivax* vir genes (Vir14, Vir2/15, Vir25), full-length PvMSP-5, Pv200L (PvMSP-1_121–416_), PvMSP-1_19_, PvDBP (RII), PvCSP-N, PvCSP-C, PvCSP-R, full-length PvCSP. Antibody data was not available for the exposed non-pregnant group (ENP).

### Cellular assays

B cell subsets were measured from PBMC samples on a BD LSR Fortessa cytometer as part of a previous study [6] and data were analysed using FlowJo software (Tree Star). B cell subsets were determined according to CD19^+^ expression and a dump channel containing a viability marker, CD3, CD14 and CD16. A Boolean gate containing live B cells and not CD10^+^ was used to gate mature viable B cells (VBCs), which were further divided into IgD switched or unswitched populations and plasma cells (IgD^−^CD38 ^high^). Furthermore, based on the expression of CD21 and CD27, switched and unswitched VBC populations were subdivided in naïve B cells (IgD^+^CD27^−^CD21^+^), MZ-like MBC (IgD^+^CD27^+^CD21^+^), active (IgD^−^CD27^+^CD21^−^) and resting (IgD^−^CD27^+^CD21^+^) classical MBC, and active (IgD^−^CD27^−^CD21^−^) and resting (IgD^−^CD27^−^CD21^+^) atypical MBC [6].

### Statistical methods

Eotaxin concentrations and anti-malaria IgG levels were not normally distributed, therefore non-parametric statistical tests were used and the variables were log_10_-transformed for regression analyses. Correlations of eotaxin-2 and eotaxin-3 concentrations with eotaxin-1 concentrations, antibody levels or cellular subset frequencies were examined with the Spearman’s correlation test. Results of correlations were interpreted according to the absolute value of rho, as follows: |0–0.19|, ‘very weak’; |0.20–0.39|, ‘weak’; |0.40–0.59|, ‘moderate’; |0.60–0.79|, ‘strong’; |0.8–1|, ‘very strong’. The Kruskal–Wallis and Mann Whitney tests were used to compare eotaxin concentrations between groups, followed by a Bonferroni Pairwise comparison to adjust the p-values for multiple testing.

Univariable linear regression models were performed to assess the associations between eotaxin-2 and eotaxin-3 concentrations (dependent variable) and *Plasmodium* infection, gestational age, age and haemoglobin levels (independent variables) for the malaria-exposed group. Models of eotaxin-2 or -3 and the variable *Plasmodium* infection were adjusted by gestational age when the latter was found to be significantly associated with eotaxin-2 or -3 in univariable models, respectively.

Pairwise Wilcoxon test was performed to compare the eotaxin-2 concentrations between peripheral and placental matched plasmas, in malaria-exposed and non-exposed pregnant women. The Mann–Whitney test was used to compare the placental eotaxin-2 concentrations between malaria-exposed and non-exposed groups. The p-values < 0.05 were considered statistically significant and p-values < 0.1 were considered a trend. Missing data for any of the variables were excluded from the analysis. R Software (Version 3.6.1 [29]) was used for the statistical analysis and graphical representation as well as the packages dplyr [30], ggplot2 [31] and rstatix [32].

## Results

### Study population

Table 1 shows the characteristics of the participants involved in the study. Median age was highest for NNP (median [IQR] of 36 [29; 44] years) and lowest for EP (25 [18; 32] years) and there were males only in the NNP group. Most women in the EP group were multigravida. All samples in the NP group were collected at delivery, and the gestational age from NP women was unknown.

**Table 1:** Description of the study participants

	ENP	EP	NNP	NP
N	38	145	23	16
Age, years median (IQR)	26 (20; 29)	25 (18; 32)	36 (29; 44)	29 (26; 32)
Sex (females), n (%)	38 (100)	145 (100)	9 (39.1)	16 (100)
*Plasmodium* infection in peripheral blood, n (%)	3 (7.69)	20 (13.8)	na	na
Gravidity, n (%)	na	Primigravida 53 (36.8)	na	nd
		1–3 pregnancies 30 (20.8)		
		4 + pregnancies 61 (42.4)		
Sampling timepoint, n (%)				
Recruitment (peripheral blood)	na	54 (37.3)	na	0 (0)
Delivery (peripheral and placental blood)	na	91 (62.7)	na	16 (100)
Gestational age by timepoint, weeks median (IQR)				
Recruitment	na	21 (12; 25)	na	nd
Delivery	na	39 (35; 43)	na	nd

*ENP* exposed non-pregnant, *EP* exposed pregnant, *NNP* naïve non-pregnant, *NP* naïve pregnant, *na* not applicable, *nd* not determined, *IQR* Interquartile range

### Correlations of peripheral plasma concentrations of eotaxin-1, eotaxin-2 and eotaxin-3

**Fig. 1:**
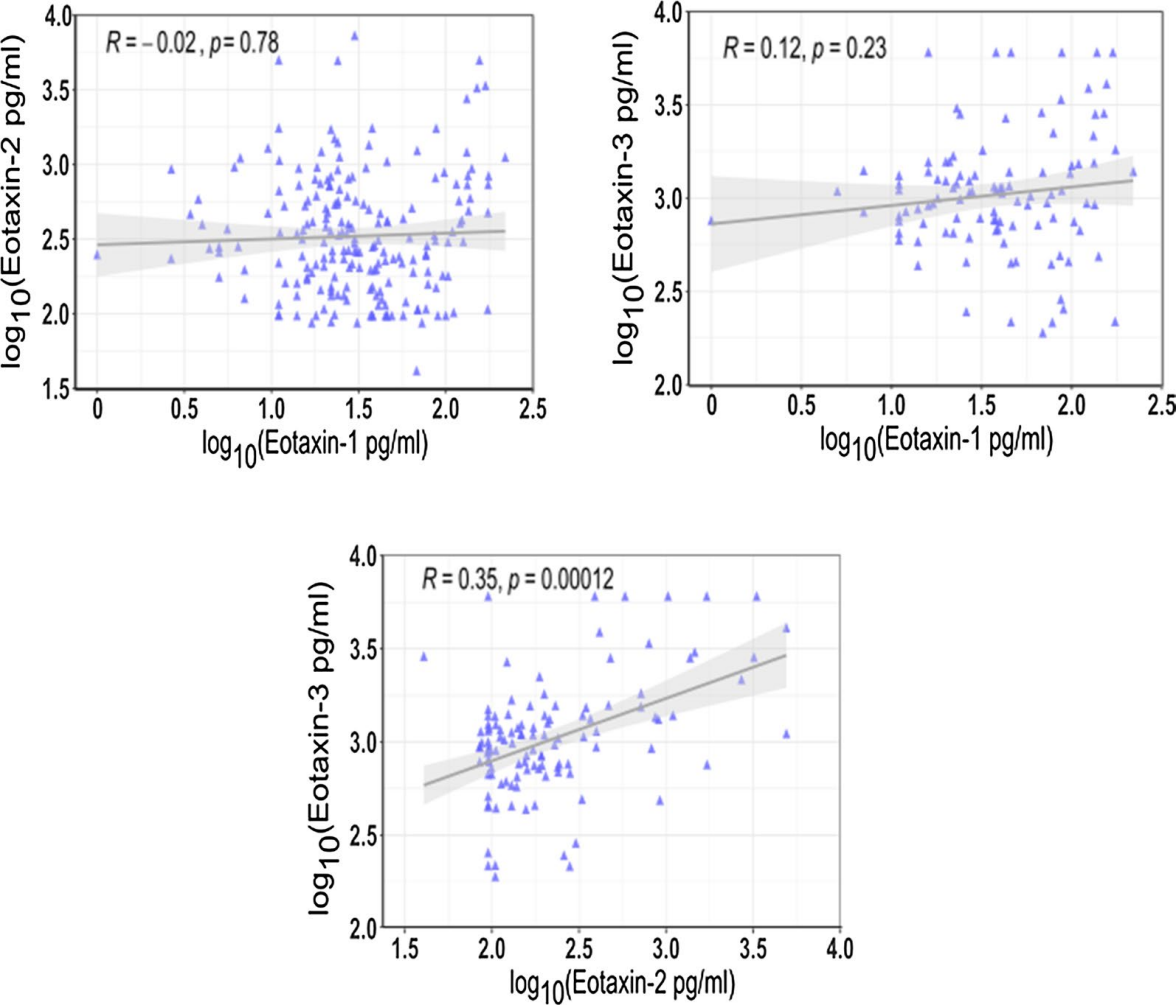
Correlations between eotaxin-1, -2 and -3 plasma concentrations. Scatter plots showing the distribution of plasma eotaxins concentrations in all study subjects, n_Eo2,Eo1_ = 202, n_Eo3_ = 116. The p-value corresponds to Spearman’s correlation test and R corresponds to the Spearman’s coefficient

There was no correlation of eotaxin-1 with eotaxin-2 or eotaxin-3 levels in the EP group. Eotaxin-2 and eotaxin-3 presented a positive and significant correlation (Spearman’s rho (R) = 0.35, p < 0.001) (Fig. 1).

### Differences in eotaxin-2 and -3 peripheral plasma concentrations by malaria exposure and pregnancy

**Fig. 2:**
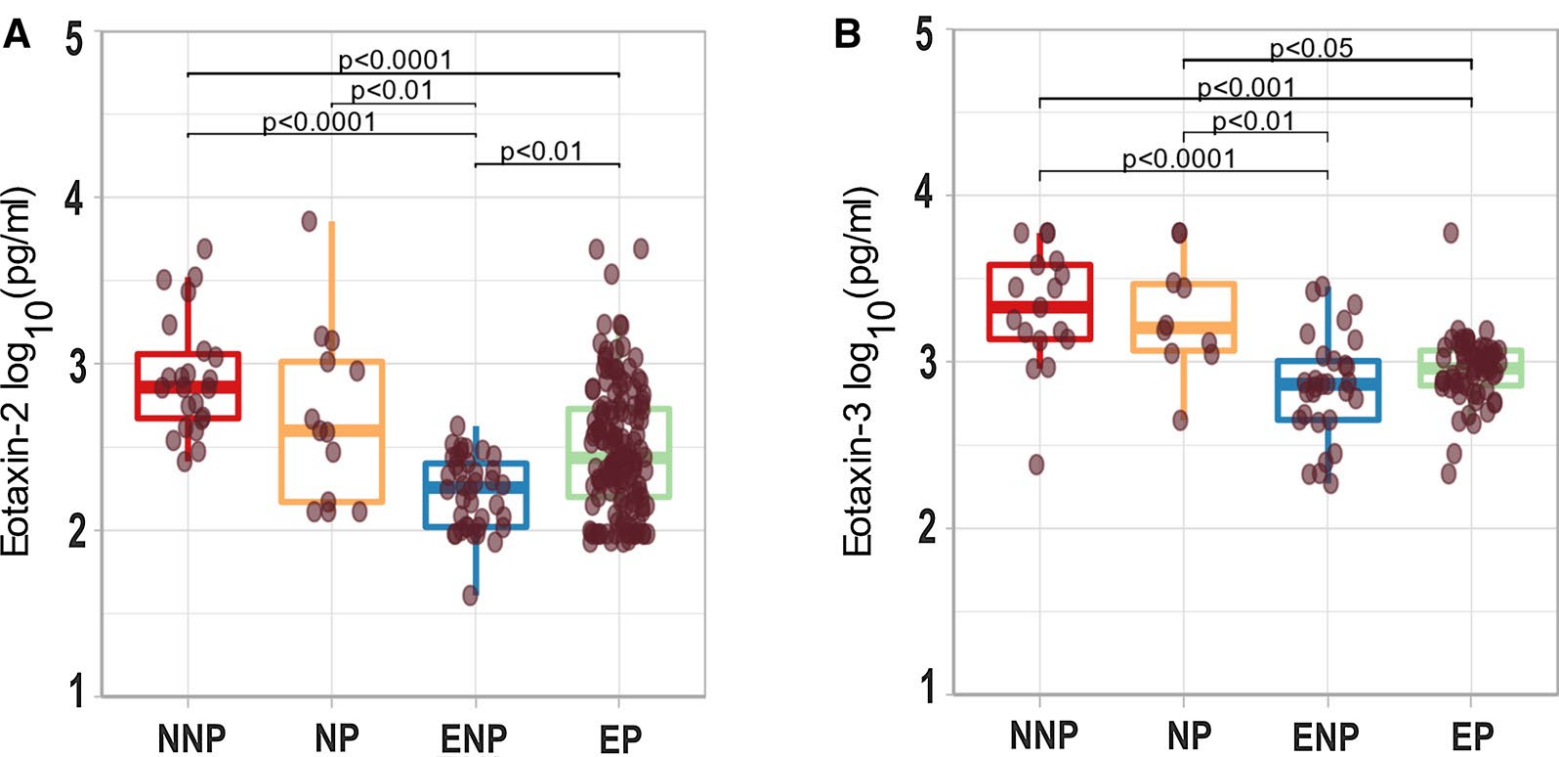
Differences in eotaxin-2 and -3 peripheral plasma concentrations by malaria exposure and pregnancy. Comparison of eotaxin-2 and eotaxin-3 plasma concentrations in malaria exposed pregnant (EP, n_Eo2_ = 145, n_Eo3_ = 60), exposed nonpregnant (ENP, n_Eo2_ = 38, n_Eo3_ = 29), non-exposed pregnant (NP, n_Eo2_ = 16 n_Eo3_ = 10) and non-pregnant (NNP, n_Eo2_ = 23, n_Eo3_ = 17) individuals. The dotplots represent the eotaxin concentrations measured in each sample. The outlayers are not shown. The boxplots correspond to the median and the 25th and 75th quartiles. The Mann Whitney test was performed for the two by two groups comparisons. P-values were adjusted for multiple comparisons using Bonferroni method. Only significant p-values are shown

There were significant differences in the eotaxin-2 concentrations between groups (p ≤ 0.001). When groups were compared two-by-two, there were not statistical differences in the plasma concentrations associated to pregnancy in the malaria-naive group (NNP vs NP), but in the malaria-exposed groups, the ENP had lower levels than the EP group (Fig. 2A). With regards to the effect of malaria exposure, an effect was found only in the non-pregnant groups. Thus, significantly lower eotaxin-2 plasma concentrations were observed in the ENP compared to the NNP group, but no differences were observed between the two pregnant groups (Fig. 2A).

With regards to eotaxin-3, there were not statistical differences in the plasma concentrations associated to pregnancy (NNP vs NP or ENP vs EP, Fig. 2B). However, significantly lower eotaxin-3 plasma concentrations were observed in malaria-exposed groups, both in the non-pregnant (NNP vs ENP) and the pregnant (NP vs EP) individuals (Fig. 2B).

### Correlations of eotaxin-2 and eotaxin-3 peripheral plasma concentrations with markers of malaria exposure

**Fig. 3:**
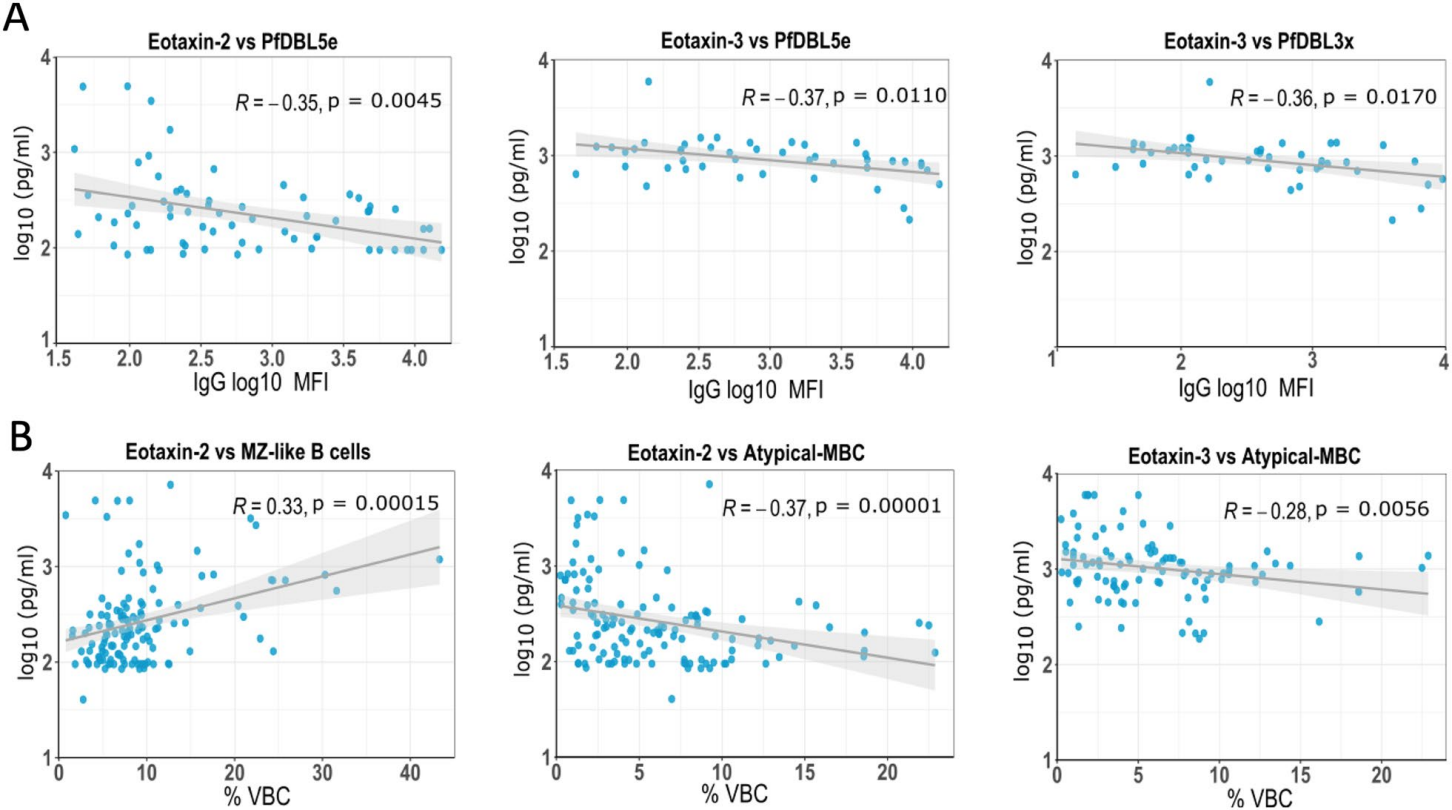
Scatterplot correlations between eotaxin-2 and -3 peripheral plasma levels and: **A** levels of IgG against PfDBL5ε and PfDBL3x; **B** frequencies of atypical MBCs and MZ-like MBCs. The p-values and the Rho coefficient (R) were obtained by a Spearman’s correlation test. *MBC* memory B cells, *MZ* marginal-zone, *R* rho Spearman’s coefficient, *VBC* viable B cells, *MFI* mean fluorescence intensity

To further investigate the association between eotaxin-2 and -3 and *Plasmodium* exposure (*P. falciparum* or *P. vivax)*, the correlations between the concentrations of these two eotaxins and IgG levels against 19 malaria antigens in peripheral plasma were assessed in the malaria-exposed group (Table 2). Most of the IgGs analysed are known markers of malaria exposure (like IgG to PfAMA-1) or MiP (like IgG to PfDBL5e or PfDBL3x). The greatest correlations observed were: PfDBL5e-IgG with eotaxin-2 and -3 (R_Eo2_ =− 0.35, p = 0.005; R_Eo3_ =− 0.37, p = 0.011) and PfDBL3x-IgG with eotaxin-3 (R_Eo3_ =− 0.36 p = 0.011) (Table 2). Each of these associations are shown as scatterplots in Fig. 3.

**Table 2:** Correlations of eotaxin-2 and eotaxin-3 peripheral plasma concentrations with malaria IgG levels

	Eo2	Eo3
PfMSP-1_19_	0	0.04
PfEBA	0.08	0.05
PfDBL6ε	− 0.23	− 0.04
PfDBL5ε	**− 0.35**	**− 0.37**
PfDBL3x	− 0.19	**− 0.36**
PfAMA-1	0.02	− 0.19
PvLP2	− 0.02	− 0.01
PvLP1	0.06	− 0.11
Vir14	0	− 0.07
Vir2/15	0.04	− 0.04
Vir25	0.09	− 0.1
PvMSP-5	0.21	0
Pv200L	− 0.14	− 0.02
PvMSP-1_19_	− 0.08	− 0.1
PvDBP	− 0.01	0.02
PvCSP-R	0.2	0.19
PvCSP-N	− 0.14	0
PvCSP-C	0.06	0.11
PvCSP	− 0.11	0.02

*The specific Spearman’s coefficient is displayed in the cells. Only the individuals of malaria exposed pregnant study group who had antibody data available are included in the analysis (EP, n*_*Eo2*_ = *85, n*_*Eo3*_ = *56). In bold if p* < *0.05. Eo Eotaxin*

Next, the correlation between eotaxin concentrations in peripheral plasma and the frequency of some B cell subsets, also known to be altered in malaria-exposed individuals and analysed in the same women as part of a previous study [6], were investigated. There was a significant negative correlation between the frequency of atypical MBC and the concentrations of both chemokines, especially eotaxin-2 (Table 3). A weaker but significant negative correlation was also observed between eotaxin-2 concentrations and active classical MBCs. In contrast, MZ-MBCs showed a positive and significant correlation with plasma concentrations of eotaxin-2. A scatter plot showing each of the strongest correlations is shown in Fig. 3.

**Table 3:** Correlations of eotaxin-2 and eotaxin-3 peripheral plasma concentrations with B cell subsets frequencies

	Eo2	Eo3
MZ-MBC	**0.33**	0.1
Naïve	0.03	0.08
rcMBC	0.09	− 0.01
aaMBC	**− 0.37**	**− 0.28**
acMBC	− 0.21	− 0.07

The specific Spearman’s coefficient is displayed in the cells. Individuals of four study groups that had available B cell data are included: NNP (n_Eo2_ = 23, n_Eo3_ = 17), NP (n_Eo2_ = 13, n_Eo3_ = 10), ENP (n_Eo2_ = 38, n_Eo3_ = 29) and EP (n_Eo2_ = 82, n_Eo3_ = 60). In bold if p < 0.05.

*MBC* memory B cells, *MZ-MBC* marginal zone-like MBC, *rcMBC* resting classical MBC, *aaMBC* active atypical MBC, *acMBC* active classical MBC, *Eo* Eotaxin

### Associations between eotaxin-2 and eotaxin-3 peripheral plasma concentrations and malaria infection

Regression models were estimated to evaluate the possible associations of eotaxin-2 and eotaxin-3 plasma concentrations with *Plasmodium* infection (*P. falciparum or P. vivax*), age, hemoglobin levels and gestational age (in the pregnant study groups). Neither age nor hemoglobin levels had an association or trend with eotaxin-2 or -3 concentrations (data not shown), but gestational age was significantly associated with eotaxin-3 concentrations being higher at delivery (Table 4*, Coeff* = *3.161; 95%CI 0.322; 6.000, p* = *0.031)* and with eotaxin-2 when recruitment and delivery samples were analyzed together *(Coeff* = *0.520; 95%CI 0.199;0.842, p* = *0.002).* Interestingly, in the exposed pregnant women, eotaxin-3 showed a trend of negative association with *Plasmodium* infection at recruitment in the crude analysis and after adjusting by gestational age (Table 4*,* adjusted effect estimate, *Coeff* = *− 0.279; 95%CI* − 0.605–0.047*, p* = *0.091*), but no association was observed at delivery. Because the prevalence of infection was relatively low at recruitment and delivery (14.19%), crude and adjusted regression models were also estimated with both pregnant groups together, but no effect was seen (Table 4).

**Table 4:** Associations between plasma eotaxin-2 and eotaxin-3 concentrations and gestational age *or Plasmodium infection*

	Eotaxin-2			Eotaxin-3		
	Coefficient	95% CI	p-value	Coefficient	95% CI	p-value
Gestational age						
Recruitment	0.159	− 0.343; 0.662	0.526	0.064	− 0.229; 0.357	0.662
Delivery	− 0.038	− 1.264; 1.187	0.950	3.161	0.322; 6.000	**0.031**
EP	0.520	0.199; 0.842	**0.002**	0.063	− 0.198; 0.326	0.629
Plasmodium infection						
Recruitment	− 0.218	− 0.822; 0.386	0.471	− 0.283	− 0.601; 0.035	**0.079**
Delivery	− 0.423	− 0.964; 0.118	0.123	− 0.256	− 0.935; 0.424	0.44
EP	− 0.296	− 0.720; 0.129	0.170	− 0.147	− 0.570; 0.284	0.49
Recruitment^a^	− 0.202	− 0.813; 0.409	0.509	− 0.279	− 0.605; 0.047	**0.091**
Delivery^a^	− 0.399	− 0.963; 0.164	0.161	− 0.454	− 1.057; 0.149	0.130
EP^a^	− 0.284	− 0.698; 0.129	0.176	− 0.121	− 0.576; 0.333	0.588

Crude or adjusted by gestational age (^a^) linear regression models were estimated with eotaxin-2 and eotaxin-3 as dependent variables and gestational age or *Plasmodium* spp. infection as independent variables, for the malaria exposed pregnant women group EP (n_Eo2_ = 145, n_Eo3_ = 60), including recruitment (n_Eo2_ = 54, n_Eo3_ = 40) and delivery (n_Eo2_ = 91, n_Eo3_ = 20) timepoints. *Plasmodium* infection included *P. vivax* or *P. falciparum* infections. Bold = p-value < 0.1

### Eotaxin-2 concentrations in peripheral and placental plasma in malaria exposed and non-exposed pregnant women

**Fig. 4:**
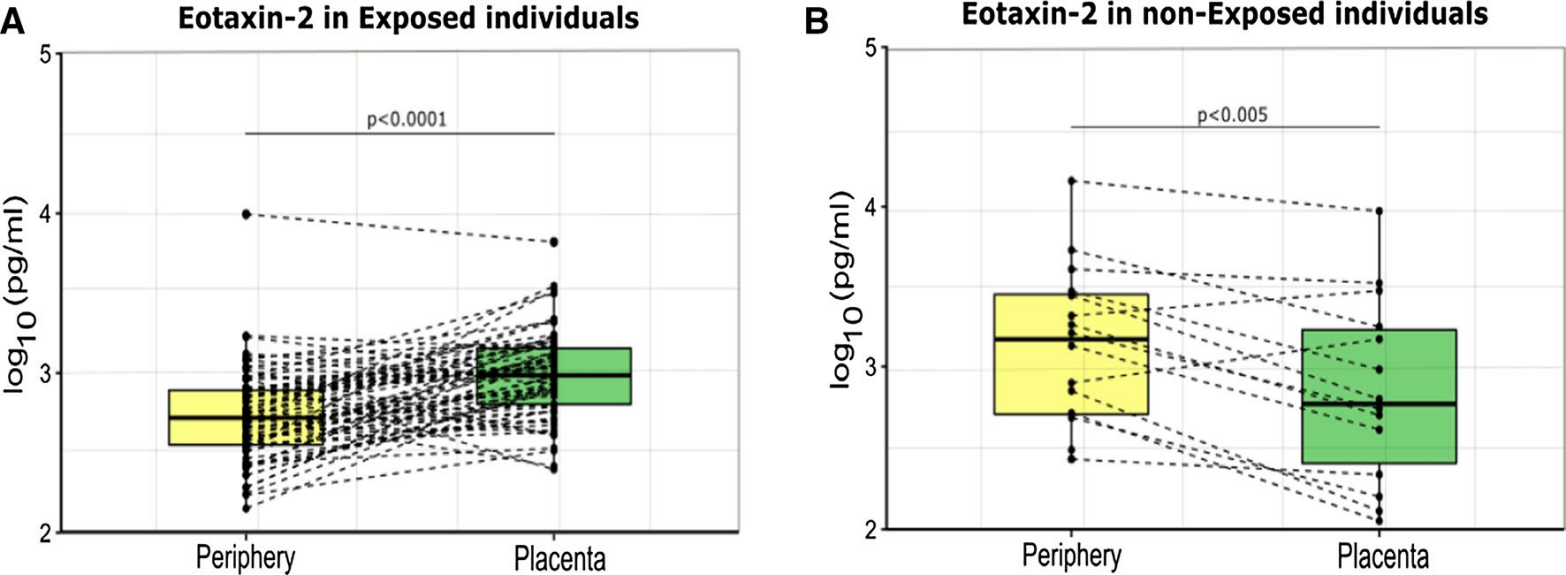
Comparison of eotaxin-2 concentrations in peripheral and placenta plasma samples. A Wilcoxon signed-rank test was performed for the comparison between peripheral and placental plasma eotaxin-2 levels in paired samples from: **A** malaria-exposed (n = 72) and **B** malaria-naive (n = 16) pregnant women. The dotplots represent the eotaxin concentrations measured in each sample, and the dotted lines connect data from paired samples. The boxplots correspond to the median and the 25th and 75th quartiles

**Fig. 5:**
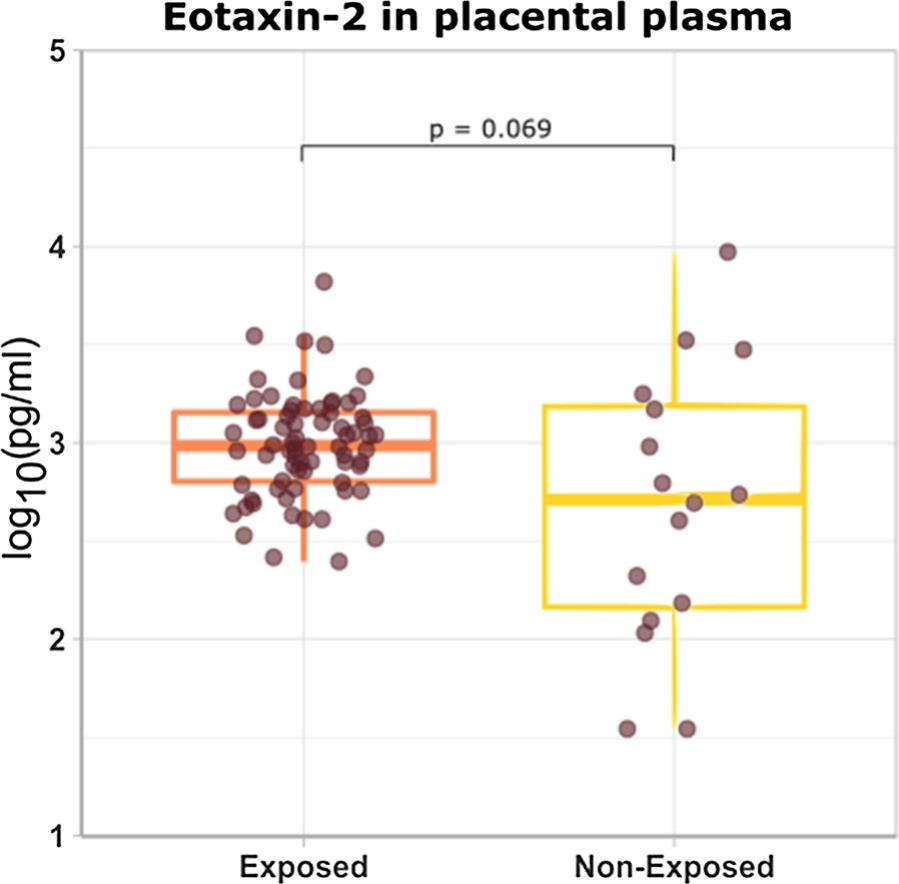
Comparison of eotaxin-2 concentrations in placental plasma from malaria exposed and non-exposed mothers. A Mann–Whitney test was performed for the comparison of eotaxin-2 placental plasma concentrations between malaria exposed pregnant women (EP-p, n = 72) and non-exposed pregnant women (NP-p, n = 16). The dotplots represent the eotaxin-2 concentrations measured in each sample. The boxplots correspond to the median and the 25th and 75th quartiles

Placental eotaxin-1 was hypothesized to be decreased in placenta compared to periphery in a tropical cohort of pregnant women [22] to avoid receptor competition with eotaxin-2, shown to enhance decidualization in vitro [33]*.* So eotaxin-2 levels should be increased in the placenta. This hypothesis was reinforced by the fact that eotaxin-2 expression in the trophoblast is 3 times bigger than the expression of eotaxin-1 or eotaxin-3 [34]. Thus, eotaxin-2 levels in placental and peripheral plasma samples were compared separately in malaria-exposed and malaria-naive women. An important and significant increase in eotaxin-2 placental plasma concentrations compared with peripheral plasma concentrations was found in malaria-exposed women (Fig. 4A), while a decrease was observed in malaria-naive women (Fig. 4B). Also, there was a trend for increased eotaxin-2 levels in placental plasma samples of malaria-exposed women compared with malaria-naïve (Fig. 5).

## Discussion

In this study, the associations between malaria exposure/infection, pregnancy and the concentrations of eotaxin-2 and 3 were investigated. In non-pregnant participants, a significant decrease of the levels of eotaxin-2 and eotaxin-3 was detected in malaria-exposed groups compared with non-exposed groups; in the pregnant women, this difference was only observed for eotaxin-3. In addition, eotaxin-2 and eotaxin-3 concentrations were significantly and inversely correlated with some *Plasmodium*-specific IgG antibodies, i.e., PfDBL5e or PfDBL3x, as well as with atypical MBC, all of them considered markers of MiP [6]. In contrast, MZ-like B cells known to be diminished in malaria-exposed individuals showed a positive and significant correlation with eotaxin-2. Although these correlations were considered to be weak, the consistency of the results make us believe that malaria exposure has a negative association with eotaxin-2 and eotaxin-3 plasma concentrations, as previously observed with eotaxin-1 plasma levels ([6], unpublished results). Of note, other infections like tuberculosis or dengue have been associated with increased and not decreased eotaxins peripheral levels [18–20]. Further explanatory studies are necessary to unravel the causes of this phenomenon, i.e. to identify the cellular source of the three eotaxins after/during malaria infection.

Also, the association of current *Plasmodium* infection and not just previous exposure with eotaxin plasma concentrations in the EP group was analysed, as *P. vivax*-infection during pregnancy was previously shown to be related to lower levels of eotaxin-1 [15]. Because in this cohort there were very few infected individuals, *P. falciparum* and *P. vivax* infection could not be analysed separately. Eotaxin-3 plasma levels at recruitment showed a negative association with *Plasmodium* infection. The association was significant even after adjusting the model by gestational age. Interestingly, a negative association of eotaxin-2 with *Plasmodium* infection could not be found in this cohort of pregnant women, despite having found moderate correlations with anti-*Plasmodium* antibody levels, and atypical and MZ-like MBCs. Lack of statistical power may have limited the detection of associations of eotaxin-2 with infection. However, it should be noted that differences in the eotaxin-2 concentrations between the EP and NP groups were not found either. Altogether these results suggest that pregnancy may modify the effect of malaria on eotaxin-2 concentrations.

With regards to the association of eotaxins with pregnancy, there were no significant differences in eotaxin-3 concentrations between pregnant and non-pregnant individuals. In the case of eotaxin-2, the concentrations in the EP group were higher than in the ENP one, in contrast to eotaxin-1, for which a pregnancy-associated decrease of levels had been observed [6]. In line with this last result, eotaxin-2 placental concentrations were higher than the peripheral ones in the malaria-exposed cohort, while eotaxin-1 followed exactly the opposite direction [15]. Moreover, eotaxin-2 placental levels were higher (although not statistically significant) in the malaria-exposed pregnant women compared to the malaria non-exposed pregnant ones. These results suggest again that eotaxin-1 and eotaxin-2 follow specific regulations in MiP. Thus, although in vitro studies have shown that the three eotaxins enhance the extravillous trophoblasts function [35], the effect of malaria infection and placental malaria on this function has yet to be determined.

This study presents some limitations. First, the measurements related to cells are not absolute counts but frequencies (proportions), thus some caution must be taken when interpreting the data. Second, cell samples were taken only from peripheral blood limiting the view of the immune response, therefore, what is seen as expansion/reduction could be a consequence of redistribution to other tissues. Third, the sample size was somehow small, especially for eotaxin-3 due to sample-volume limitations, limiting the power to detect statistical associations particularly with infection in adjusted models. Fourth, although samples from non-pregnant individuals were used as controls, a cohort of *Plasmodium*-infected non-pregnant individuals to ascertain the effect of malaria in eotaxin-2 and eotaxin-3 levels would be necessary.

## Conclusion

Eotaxin-3 is inversely associated with malaria exposure and infection during pregnancy, as seen previously with eotaxin 1. Eotaxin-2 concentrations show an opposite pattern to eotaxin-1 in MiP, with increased levels in pregnant compared to non-pregnant malaria-exposed groups, increased placental levels compared to periphery, and enhanced placental concentrations in the malaria-exposed than in the malaria-naive women. More research on the implication of all eotaxins in malaria and its interaction with pregnancy is required to elucidate the particular role of each of them and to clarify the immune mechanism behind their associations with B cell subsets.

## Data Availability

The datasets used and/or analyzed during the current study are available from the corresponding author on reasonable request.

## Abbreviations

AMA1: Apical Membrane Antigen 1
CCR: C–C chemokine receptor
CD: Cluster of differentiation
CI: Confidence interval
CSP: Circumsporozoite protein
DBL: Duffy-Binding-like
DBP: Duffy binding protein
EBA: Erythrocyte binding antigen
ELISA: Enzyme-linked immunosorbent assay
ENP: Exposed Non-Pregnant
EP: Exposed pregnant
EP-d: Exposed pregnant at delivery
EP-e: Exposed pregnant at enrolment
EP-p: Placental plasma of exposed pregnant
HIV: Human immunodeficiency virus
IQR: Interquartile range
LP1/LP2: Long synthetic peptides containing conserved VIR sequences
MBC: Memory B cells
MFI: Mean fluorescence intensity
MiP: Malaria infection during pregnancy
MSP: Merozoite surface protein
MZ: Marginal zone
NAI: Naturally acquired immunity
NK cell: Natural killer cells
NNP: Non-exposed Non-Pregnant
NP: Non-exposed pregnant
PBMC: Peripheral blood mononuclear cells
PCR: Polymerase chain reaction
PNG: Papua New Guinea
Pv200L: Plasmodium vivax MSP-1 fragment 200L
R: Rho
RBC: Red Blood Cells
VAR2CSA: Variant surface antigen 2-CSA
VBC: Viable B cells
VCAM: Vascular cell adhesion protein 1
Vir: Antibody against VIR proteins
WHO: World Health Organization
